# Gender Differences in Sleep Problems Among Drug Users

**DOI:** 10.3389/fpsyt.2020.00808

**Published:** 2020-08-12

**Authors:** Haoyu He, Jinsong Tang, Tieqiao Liu, Wei Hao, Yanhui Liao

**Affiliations:** ^1^Department of Psychiatry, The Second Xiangya Hospital, Central South University, Changsha, China; ^2^National Clinical Research Center on Mental Disorders, Changsha, China; ^3^Department of Psychiatry, School of Medicine, Sir Run Run Shaw Hospital, Zhejiang University, Hangzhou, China; ^4^Key Laboratory of Medical Neurobiology of Zhejiang Province, Hangzhou, China

**Keywords:** sleep quality, sleep problems, drug use, healthy control, gender differences

## Abstract

**Background:**

Illicit drug use has been recognized as a major problem. Clinical studies demonstrated that poor sleep quality was associated with increased frequency of drug use and relapse. However, few studies addressed the issue of sleep quality and gender differences in illicit drug dependent subjects. The present study aimed to explore the gender differences in sleep problems in drug users.

**Methods:**

In this cross-sectional study, a total of 2,178 illicit drug users, including 1,875 male users [884 methamphetamine (MA) users and 991 heroin or other drug users] and 303 female users (78 MA users and 225 heroin or other drug users, 13.9%), from drug rehabilitation centers in Changsha, and 2,236 non-drug-using subjects, including 1,910 males and 326 females (14.6%) completed the self-report Pittsburgh Sleep Quality Index (PSQI).

**Results:**

We found that the prevalence of suggestive sleep problems (PSQI>5) between male and female was 67.4 and 75.2% in overall illicit drug use sample (p<0.001), 52.4 and 75.6% in MA users (<0.001), 80.8 and 75.1% in heroin or other drugs users (p=0.054), 26.0 and 28.8% in healthy controls (p=0.287). For sleep quality, the mean of PSQI total score (M±SD) between male and female was 7.8±4.42 and 8.9±4.15 in overall illicit drug users (p<0.001), 6.4±4.45 and 9.1±4.00 in MA users (<0.001), 9.1±3.96 and 8.9±4.21 in heroin or other drugs users (p=0.394), 4.2±2.46 and 4.4±2.51 in healthy control sample (p=0.090). These results indicated that only MA users, rather than heroin or other drugs users and healthy controls, showed gender differences in sleep problems.

**Conclusions:**

In this study, female users reported higher frequency of sleep problems and poorer sleep quality than male users in MA users’ group, but not in heroin or other drug users group. Future studies aim at quantifying the benefits of treatment interventions should not neglect the influence of sleeping problems and its gender differences.

## Introduction

Sleep disorders are prevalent problems in the general population and are closely connected with medical, psychological, and social disturbances (1). An early study from the Los Angeles metropolitan area showed that approximately half of people from general population displayed sleep disorders (2). Insomnia is the most common sleep disorder. It is characterized by the presence of an individual’s report of difficulty falling and/or staying asleep. About one-third of the adult general population experience one or more of the symptoms of insomnia, such as difficulty initiating sleep, difficulty maintaining sleep, waking up too early, and nonrestorative or poor quality of sleep (3, 4).

Insomnia is associated with substantial impairments in emotional and mental health, and quality of life (5, 6). Sleep problems are much more likely to affect patients with mental disorders, such as anxiety, depression, bipolar disorder, and attention deficit hyperactivity disorder (ADHD), alcohol, and other substance use disorders, than people in the general population (7–9).

Sleep problems may increase risk for developing substance use disorders, as well as result from chronic substance use. An ongoing longitudinal study showed that early childhood sleep problems (3–5 years of age) significantly increased the likelihood of an early onset of any use of alcohol and illicit drugs, as well as an early onset of occasional or regular cigarette smoking in adolescence (12–14 years of age) (7). Heroin and methamphetamine (MA) are the most commonly used illicit drug in China (10). Our previous study showed that a majority of heroin users displayed poor sleep quality, especially those long-term users (11). Other chronic drug users [such as MA users and ketamine users (12)], and individuals with behavior addiction [such as Internet addiction (13) and Instagram addiction (14)] were also reported high prevalence of sleep problems. The illicit drugs, such as methamphetamine, cocaine, ecstasy, and marijuana, have pronounced impact on sleep quality by polysomnography (PSG, a test that electronically transmits and records specific physical activities while in sleep). For example, cocaine administration suppresses rapid eye movement (REM) sleep and increases wakefulness; abstinent heavy 3,4-methylenedioxymethamphetamine (MDMA; “ecstasy”) users display altered sleep architecture (15).

It is quite evident that sleep problems are more common in women than in men in the general population (16, 17). Our previous study with a large sample from the general population in China also shows that women reported poorer sleep quality than men did, but little gender difference in the prevalence of insomnia (4). However, gender differences of sleep problems were found to be associated with socio-economic patterning, people with low income, low education have more chance to suffer from sleep problems (17). Our previous study found that smokers, especially heavy smokers had more sleep problems than nonsmokers (18). However, gender differences of sleep problems among drug users, especially the mainly used MA and heroin, are not fully understood.

The mechanism of action of MA involves blockade of presynaptic reuptake and displacement of vesicular stores of various neurotransmitters, mainly affected dopamine, norepinephrine, and serotonin. Central nervous system effects of acute MA abuse include insomnia, anxiety, tremors, hallucinations, paranoia, chronic effects include paranoia, hallucinations, paranoid psychosis, memory loss, depression (19). Heroin is an opioid (narcotic) drug made from morphine. Acute heroin abstinence changes various states of sleep, waking and related behaviors (20), causing sedation and daytime drowsiness (side effects of opioid analgesics) (21). Chronic heroin use also linked to poor sleep quality (11, 22).

The present study sought to explore them in a sample of drug users and healthy controls. It is hypothesized that the female drug users, either MA users or heroin or other drug users, would report more sleep problems and poorer sleep quality than the male ones. It is also hypothesized that, compared with healthy controls, gender differences of sleep problems and poorer sleep quality would be more significant in drug users.

## Methods

### Subjects

A total of 2,390 healthy nondrug users and a total of 2,178 illicit drug users (after detoxification-treatment) from voluntary drug rehabilitation centers (the Kangda Voluntary Drug Rehabilitation Centre in Hunan Province and the Department of Addiction Medicine, Hunan Brain Hospital) in Changsha completed the self-report Pittsburgh Sleep Quality Index (PSQI) to explore sleep quality among them. The data of drug users was collected after their completion of a 10-day detoxification-treatment. The date of healthy controls was collected by visiting demographically matched households. Healthy controls were excluded if they reported any drug use, major medical, or psychiatric disorders. More details about subjects see (12).

### Assessment Measures

Socio-demographic and sleep-related information were gathered for all participants. Drug used related information were collected for all drug users. The Chinese version of Pittsburgh Sleep Quality Index (PSQI) (23) was used to assess sleep quality and sleep problems. PSQI total score > 5 was an indicator of suggestive poor quality of sleep (poor sleeper).

### Procedure

The study and oral informed consent were approved from the Second Xiangya Hospital of Central South University Institutional Review Board (No. S117, 2009). All participants were informed that they can withdraw from the study at any point without penalty or adverse consequences. Issues of confidentiality and anonymity were discussed with them. Oral informed consent was obtained from all participants. Drug dependent patients were invited to answer the short self-reported questionnaire on the first week of the administration about their drug use. Eligible participants were invited to participant the survey after their completion of a 10-day detoxification-treatment.

### Statistical Analysis

Statistical analysis was performed using the SPSS for Windows (Version 22, SPSS Inc., Chicago, IL, USA) software package. Descriptive statistics were used to examine demographic and drug use characteristics, and pooled responses. Mann-Whitney U-test and χ^2^ (Chi-square) were conducted to determine gender differences of demographic characteristics, sleep problems (including subjective sleep quality, sleep latency, sleep duration, habitual sleep efficiency, sleep disturbance, need for sleep medications, daytime dysfunction), sleep quality (by the mean of PSQI total score), and poor sleepers (suggestive insomnia) in each group (healthy control group, overall drug use group, MA use group, heroin, or other drugs use group). Analysis of variance (ANOVA) was applied to explore gender and group differences in sleep quality (by the mean of PSQI total score). First, one-way ANOVA was used to compare each two-group differences across three groups (healthy control group, MA use group, heroin, or other drugs use group) of males and females. Then, further explored whether there were differences by gender (male, female)/drug class (MA, heroin or other drug) differences (either main effect or an interaction with gender) with general linear model (GLM) of univariate ANOVA. A *post-hoc* test was conducted using the Fisher’s least significant difference (LSD). In addition, χ^2^ was conducted to compare the percentage of poor sleepers (suggestive insomnia with PSQI > 5) among healthy controls, heroin, or other drug users and MA users in samples of overall, male, and female, respectively. A p-value threshold of 0.01 was set to determine statistical significance.

## Results

Demographic characteristics between male and female in healthy controls and drug users (MA users and heroin or other drug users) are shown in **Table 1**. Cigarette smoking and alcohol drinking are the most significantly different characteristics between males and females in control group. Only a very small proportion of healthy females were cigarette smokers (1.9%) or alcohol drinkers (2.8), but approximately 90% and over 10% of female drug users were cigarette smokers and alcohol drinkers, respectively. The MA users used an average of 36 (mean ± SD: 36.2 ± 31.44) months of MA, and heroin users 82 (82.1 ± 60.81) months of heroin. In the last 3 months before administration, MA users used an average of 0.6 g (0.6 ± 0.91) MA with an average of two (2.3 ± 1.13) times per day, heroin users used 1.7 g (1.7 ± 0.98) heroin with an average of one (1.1 ± 0.34) time per day.

**Table 1:** Demographic characteristics between male and female in groups of healthy controls and drug users.

Variables	Healthy controls (n=2,236)			Drug users (n=2,178)								
				Overall sample			MA users (n=962)			Mainly heroin users^#^ (n=1,216)		
	Male(n=1,910)	Female(n=326, 14.6%)	p*	Male(n=1,875)	Female(n=303, 13.9%)	p*	Male(n=884)	Female(n=78, 8.4%)	p*	Male(n=991)	Female(n=225, 18.5%)	p*
Age (yrs), M±SD	30.7±11.23	30.2±10.66	0.486	30.0±7.13	30.1±7.16	0.776	29.1±6.79	26.7±5.95	0.003	30.9±7.33	31.3±7.18	0.428
Education (yrs), M±SD	12.2±3.23	11.7±3.19	0.008	10.7±2.96	10.4±3.05	0.070	11.1±2.89	10.2±2.99	0.004	10.2±2.96	10.5±3.07	0.298
Drug use duration (month), M±SD	–	–	–	59.0±51.73	62.3±55.38	0.581	36.6±32.03	31.9±23.49	0.205	78.9±57.47	72.9±59.25	0.157
Ethnicity (Han Chinese, %)	96.7%	95.1%	0.146	98.7%	99.7%	0.135	98.6%	98.7%	0.955	98.7%	100%	0.084
Employed %	62.6%	52.5%	0.001	55.1%	61.9%	0.026	47.0%	66.7%	0.001	62.2%	60.3%	0.589
Married %	50.8%	55.4%	0.125	45.2%	67.7%	<0.001	41.9%	24.4%	0.002	48.2%	35.1%	<0.001
Cigarette smoking%	46.8%	1.9%	<0.001	97.8%	90.1%	<0.001	98.0%	88.5%	<0.001	97.7%	90.7%	<0.001
Alcohol drinking%	35.7%	2.8%	<0.001	38.3%	13.9%	<0.001	53.0%	16.7%	<0.001	25.2%	12.9%	<0.001

M, mean; SD, standard deviation; n, number; %, the percentage of subjects.

Cigarette smoker was defined as smoked more than 100 cigarettes in the life time.

Alcohol drinking was defined as drunk no less than 30 g alcohol (equal to 900 ml beer) per week.

Difference in percentage or mean differences analyzed between male and female drug users by the Chi-squared test and the Wilcoxon rank sum test (Mann-Whitney U-test) respectively.

^#^Mainly heroin users (heroin or other drug users, including 1,012 heroin users in a total of 1,216 subjects, 83.2%), and some poly-substance users who mainly used “club-drug”.

*Significantly different from male group, p < 0.01.

The PSQI components’ scores and PSQI total score between male and female in healthy control group and drug use (methamphetamine, heroin, or other drugs) group are shown in **Table 2**. The prevalence of suggestive sleep problems (PSQI>5) between male and female was 67.4 and 75.2% in overall illicit drug use sample (p<0.001), 52.4 and 75.6% in MA users (<0.001), 80.8 and 75.1% in heroin or other drugs users (p=0.054), but little gender difference was found in healthy control sample (26.0% in males and 28.8% in females, p=0.287). The mean of PSQI total score (M±SD) between male and female was 7.8±4.42 and 8.9±4.15 in overall illicit drug use sample (p<0.001), 6.4±4.45 and 9.1±4.00 in MA users (<0.001), 9.1±3.96 and 8.9±4.21 in heroin or other drugs users (p=0.394), 4.2±2.46 and 4.4±2.51 in healthy control sample (p=0.090). These results indicated that only female MA users, rather than heroin or other drugs users and healthy controls, had overall poorer sleep quality or more poor sleepers than males MA users. Noticeably, all drug users, either male or female, MA users or heroin or other drug users, had significantly poorer sleep quality and more sleep problems than that in healthy controls.

**Table 2:** Score of the Pittsburgh Sleep Quality Index (PSQI) components and PSQI total score between male and female in groups of healthy controls and drug users.

	**Healthy controls (n=2,236)**			**Drug users (n=2,178)**								
				Overall sample			MA users (n=962)			Mainly heroin users (n=1,216)		
	Male	Female	P*	Male	Female	P*	Male	Female	P*	Male	Female	P*
**Subjective sleep quality, M±SD**	0.8±0.64	0.7±0.64	0.004	1.4±0.90	1.6±0.84	<0.001	1.2±0.93	1.6±0.89	<0.001	1.6±0.83	1.6±0.82	0.615
**Sleep latency, M±SD**	0.7±0.69	1.0±0.85	<0.001	1.4±1.13	1.6±1.08	0.003	1.2±1.13	1.7±1.17	<0.001	1.6±1.11	1.6±1.05	0.960
**≤15 min, %**	42.9	29.1	–	27.5	17.2	–	35.4	16.7	–	20.5	17.3	–
**16–30 min, %**	46.5	49.1	–	29.7	34.3	–	27.8	33.3	–	31.3	34.7	–
**31–60 min, %**	9.3	15.0	–	18.2	19.1	–	16.7	9.0	–	19.6	22.7	–
**>60 min, %**	1.3	6.7	–	24.6	29.4	–	20.0	41.0	–	28.7	25.3	–
**Sleep duration, M±SD**	0.8±0.82	0.5±0.74	<0.001	0.8±0.99	1.0±1.06	0.007	0.6±0.93	1.2±1.12	<0.001	1.0±1.01	0.9±1.04	0.475
**>7 h, %**	46.0	62.0	–	51.1	45.2	–	61.5	41.0	–	41.8	46.7	–
**5–7 h, %**	52.1	37.7	–	40.6	42.9	–	31.9	44.9	–	48.4	42.2	–
**<5 h, %**	1.9	0.3	–	8.3	11.9	–	6.6	14.1	–	9.9	11.1	–
Habitual sleep efficiency*, M±SD	0.3±0.70	0.4±0.62	0.168	0.6±1.03	0.8±1.14	0.023	0.5±0.92	1.0±1.24	0.001	0.8±1.10	0.7±1.10	0.621
**≥85%, %**	80.9	71.5	–	65.9	60.7	–	72.6	55.1	–	59.9	62.7	–
**75–84%, %**	12.0	23.0	–	15.3	14.2	–	13.6	12.8	–	16.8	14.7	–
**65–74%, %**	3.5	4.3	–	7.4	9.2	–	5.8	10.3	–	8.9	8.9	–
**<65%, %**	3.5	1.2	–	11.4	15.8	–	8.0	21.8	–	14.4	13.8	–
**Sleep disturbance, M±SD**	0.7±0.53	0.8±0.55	0. 003	1.1±0.72	1.2±0.79	0.028	1.0±0.70	1.4±0.70	<0.001	1.2±0.72	1.2±.81	0.181
**Need for sleep medications, M±SD**	0.1±0.27	0.1±0.34	0.271	0.9±1.23	1.0±1.30	0.151	0.6±1.07	0.7±1.19	0.663	1.1±1.32	1.1±1.33	0.963
**Not during the past month, %**	95.4	93.9	–	62.7	60.7	–	71.8	74.4	–	54.5	56.0	–
**Less than once a week, %**	3.7	5.2	–	8.4	5.0	–	8.0	2.6	–	8.8	5.8	–
**Once or twice a week, %**	0.7	0.3	–	8.3	9.6	–	7.4	5.1	–	9.2	11.1	–
**≥3 times a week, %**	0.1	0.6	–	20.6	24.8	–	12.8	17.9	–	27.5	27.1	–
**Daytime dysfunction, M±SD**	0.9±0.85	1.0±.85	0.010	1.6±1.10	1.7±1.14	0.091	1.3±1.10	1.6±1.07	0.017	1.9±1.03	1.8±1.16	0.159
**PSQI total score, M±SD**	4.2±2.46	4.4±2.51	0.090	7.8±4.42	8.9±4.15	<0.001	6.4±4.45	9.1±4.00	<0.001	9.1±3.96	8.9±4.21	0.394
**Poor sleepers (PSQI > 5), %**	26.0	28.8	0.287	67.4	75.2	<0.001	52.4	75.6	<0.001	80.8	75.1	0.054

M, mean; SD, standard deviation; n, number; %, the percentage of subjects; PSQI, Pittsburgh Sleep Quality Index.

Difference in percentage or mean differences analyzed between male and female drug users by the Chi-squared test and the Wilcoxon rank sum test (Mann-Whitney U-test) respectively.

*Significantly different from male group, p < 0.01.

The total score range ranges from 0 to 70 and each score ranges from 0 to 3, with higher scores indicating poorer functioning.

Habitual sleep efficiency^*^ = total hours of sleep/(get-up time−bedtime) × 100%.

**Table 3** showed that samples of overall, male, and female were all significantly different in sleep quality (mean of PSIQ total) among healthy controls, heroin, or other drug users and MA users (p<0.001), expect for comparing female heroin or other drug with female MA groups (p=0.61). Then, further explored whether there were differences by gender [male and female, nondrug or drug class (MA and heroin or other drug) differences (either main effect or an interaction with gender), and showed significant effects of gender (F=26.742, p<0.001)], group (F=440.705, p<0.001) and gender*group (F=19.809, p<0.001). **Table 4** showed that samples of overall, male, and female were all significantly different in poor sleeping (PSQI>5) among healthy controls, heroin, or other drug users and MA users, expect for comparing female heroin or other drug with female MA groups (p=0.926).

**Table 3:** Pittsburgh Sleep Quality Index (PSQI) total score comparisons among healthy controls, heroin users and MA users in samples of overall, male, and female, respectively.

Groups		N	Mean	SD	95% CI (lower, upper)	P*
Overall	Control	2,236	4.2	2.47	4.09, 4.30	<0.001
	MA	962	6.6	4.48	6.31, 6.88	
	Heroin	1,216	9.1	4.01	8.84, 9.29	
Male	Control	1,910	4.2	2.46	4.05, 4.27	<0.001
	MA	884	6.4	4.45	6.08, 6.67	
	Heroin	991	9.1	3.96	8.86, 9.36	
Female	Control	326	4.4	2.51	4.14, 4.68	<0.001
	MA	78	9.1^#^	4.00	8.18, 9.98	
	Heroin	225	8.9^#^	4.21	8.30, 9.40	

CI, confidence interval for mean.

*Significantly different from male group, p < 0.01.

^#^No significant differences between female heroin and female MA groups, p = 0.61.

**Table 4:** The percentage of poor sleepers [Pittsburgh Sleep Quality Index (PSQI) > 5] comparisons among healthy controls, mainly heroin users (heroin or other drug users) and MA users in samples of overall, male, and female, respectively.

	Controls	MA users	Mainly heroin users	P^*^
Overall	26.4%	54.2%	79.8%	<0.001
Male	26.0%	52.4%	80.8%	<0.001
Female	28.8%	75.6%^#^	75.1%^#^	<0.001

*Significantly different from male group, p<0.01.

^#^No significant differences between female heroin and female MA groups, p = 0.926.

## Discussion

To our knowledge, this is the first study to assess gender differences in sleep problems among illicit drug users. The current study found that gender differences in sleep problems occurred only in MA users, but not in heroin or other drug users or healthy controls.

The overall prevalence of suggestive sleep problems or disturbances (PSQI>5) between male and female was 67.4 and 75.2% in overall illicit drug use sample, with 52.4 and 75.6% in MA users, 80.8 and 75.1% in heroin or other drugs users, and 26.0% in healthy males and 28.8% in healthy females. For sleep quality, the mean of PSQI total score between male and female was 7.8 and 8.9 in overall illicit drug users, 6.4 and 9.1 in MA users, 9.1 and 8.9 in heroin or other drugs users, 4.2 and 4.4 in healthy control sample. Compared with healthy controls, all drug users, especially male heroin or other drug users, had much worse sleep quality. Our previous study already showed higher prevalence of sleep problems in drug users, especially in heroin or other drug users, than healthy controls. Furthermore, heroin or other drug users experienced more sleep problems than MA users (12). However, this study found that female MA users experienced sleep problems similar to female heroin or other drug users. Only MA users showed gender differences in sleep problems, i.e., a higher proportion of female poor sleepers.

Previous studies showed that insomnia certainly had a more profound impact on females than on males (24). The gender difference in the prevalence of sleep problems, specifically the excess in females compared with males, was also observed from our previous large sample size study (4). This study, surprisingly, showed that female drug users had poorer sleep quality than those males only in MA users. Although with no statistically significant differences, females had a trend of poorer sleep quality than their counterparts in general population, and male had a trend of poorer sleep quality than female heroin or other drug users. The underline factors that contributed to these trends are worth exploring in the future studies.

One of the interesting findings is the differences in sleep duration between male and female drug users. Our previous study with a sample (n = 26,851) from the general population found that a total of more women (60.6%) than men (55.9%) reported more than 7 h sleep duration (4). In this study, consistent with the large sample survey, we found that more females had more than 7 h sleep duration in healthy controls and heroin or other drug users, but less female MA users (41%) had more than 7 h sleep duration than male MA users (61.5%). Furthermore, compared with male MA users, more than twice of female MA users (41%) reported sleep latency with more than 1 h.

Short sleep durations have been associated with negative health outcomes (25). For example, short self-reported sleep duration was associated with the incident diagnosis of hypertension, which may result from disrupting circadian rhythmicity and autonomic balance (26). Furthermore, the self-reported sleep duration was associated with interleukin-6 (IL-6) and high-sensitivity C-reactive protein (hs-CRP) in women but not in men (27). Further studies will be needed to establish whether the connection between short sleep duration and female gender in drug use, whether this connection can be demonstrated with other measures, and whether the relationship is causal.

Gender differences in acute effects of MA on subjective mood and reward-related behavior (28) may contribute to gender differences in sleep problems, i.e., compared to their counterparts, female MA (rather than heroin or other drug) users showed overall poorer sleep quality and high prevalence of sleep problems. In addition, MA users experienced significantly more depressive symptoms than healthy controls (29), and there is a gender differences in depression-females are more likely than males to present with depression (30).

The substance/gender interactions with sleep quality could be more complicated. Even a single intranasal methamphetamine dose could produce significant reductions on measures of subjective sleep quality and objective sleep (31). The gender differences of sleep quality between male and female MA users may thank to the gender-specific contribution of the specific genes in MA use disorder (32) or due to the fact that female MA users experience higher rates of depression than their counterparts (33). Furthermore, compared with male MA users, although female users seem more dependent to MA, they show diminished dopamine responses and fewer cases of emergency department-related deaths involving MA (34). Further explore the underline factors associated with gender differences in sleep quality between male and female MA users may provide new evidence-based effective treatment for MA use disorders, especially for these female MA users.

Previous research found that, compared with no sleep disturbance, poor sleep quality or insomnia were associated with higher frequency of cocaine use (35). On the other hand, disturbed sleep and daytime sleepiness occupies in adolescence were associated substance abuse and emotional outcomes, and associated with poor addiction treatment outcomes (36). Thus, sleep quality may serve as a predictor for drug treatment and relapse. Assessing and addressing insomnia and its gender differences among treatment seeking drug users may improve treatment outcome and prevent relapse.

### Limitations

In China, men have much higher rates of use or dependence on illicit drugs than do women (37). Thus, the current sample included only 13.9% of female drug users. Furthermore, female drug users reported higher heroin use rate (18.5%) and lower MA use rate (8.4%) that male users. Another limitation is that the percentage of cigarette smoking and alcohol drinking was lower in female drug users than that in male users. However, previous studies showed that both cigarette smoking (38) and high-risk drinking behaviors (39) were more likely to report sleep problems. Furthermore, this study did not assess many other sleep problems related factors, such as socio-economic status, medicine, drug use patterns, severity of dependence. On the other hand, sleep problems in early childhood can predict the onset of alcohol-related problems and illicit drug use in adolescence (7). In the current study, even fewer women than men had cigarette smoking and alcohol drinking, they still reported more sleep problems than male MA users. In addition, this study only assessed substance use, but not internet or other types of behavior addiction, which may also associated with sleep problems (13). Some of the drug users may also try other drugs. However, this study excluded heroin users or MA users who also mainly used other drugs. Last, the minority population of LGBT (lesbian, gay, bisexual, and transgender) may have high risk of drug use (40, 41). This study, however, did not include the third gender.

## Conclusion

In conclusion, our data suggest that female MA users reported poorer sleep quality by presenting higher PSQI total score than male users. This study found no gender differences in sleep problems in heroin or other drug users or in healthy controls. Female MA users also showed shorter sleep duration than that in male users. Future studies aim at quantifying the benefits of treatment interventions for MA users should not neglect the influence of sleeping problems and its gender differences. Gaining more insight into the impact of gender differences of sleep quality on the MA addiction treatment could also help to target future intervention measures more effectively for female users.

## Data Availability Statement

The raw data supporting the conclusions of this article will be made available by the authors, without undue reservation.

## Ethics Statement

The studies involving human participants were reviewed and approved by the Second Xiangya Hospital of Central South University Institutional Review Board (No. S117, 2009). Written informed consent for participation was not required for this study in accordance with the national legislation and the institutional requirements.

## Author Contributions

YL conceived the study. YL and HH did the literature review, statistical analyses, and drafted the report. YL and HH collected the data. YL took the lead in writing the manuscript. TL and JT interpreted the data and commented on the manuscript. All authors contributed to the article and approved the submitted version.

## Funding

The study was supported by the Natural Science Foundation of China (Grant No. 81671325 to YL, 81671324 to TL). The funders had no role in study design, data collection and analysis, decision to publish, or preparation of the manuscript.

## Conflict of Interest

The authors declare that the research was conducted in the absence of any commercial or financial relationships that could be construed as a potential conflict of interest.

## References

[1] Vgontzas AN Kales A Sleep and its disorders. Annu Rev Med (1999) 50:387–400. 10.1146/annurev.med.50.1.387 10073285

[2] Bixler EO Kales A Soldatos CR Kales JD Healey S Prevalence of sleep disorders in the Los Angeles metropolitan area. Am J Psychiatry (1979) 136:1257–62. 10.1176/ajp.136.10.1257 314756

[3] Ancoli-Israel S Roth T Characteristics of insomnia in the United States: results of the 1991 National Sleep Foundation Survey. I. Sleep (1999) 22:S347–53. 10394606

[4] Tang J Liao Y Kelly BC Xie L Xiang Y-T Qi C Gender and regional differences in sleep quality and insomnia: a general population-based study in Hunan Province of China. Sci Rep (2017) 7:43690. 10.1038/srep43690 28262807 PMC5337959

[5] Roth T Insomnia: definition, prevalence, etiology, and consequences. J Clin sleep medicine: JCSM: Off Publ Am Acad Sleep Med (2007) 3:S7. 10.5664/jcsm.26929 PMC1978319 17824495

[6] Benbir G Demir AU Aksu M Ardic S Firat H Itil O Prevalence of insomnia and its clinical correlates in a general population in T urkey. Psychiatry Clin Neurosci (2015) 69:543–52. 10.1111/pcn.12252 25384688

[7] Wong MM Brower KJ Fitzgerald HE Zucker RA Sleep problems in early childhood and early onset of alcohol and other drug use in adolescence. Alcoholism: Clin Exp Res (2004) 28:578–87. 10.1097/01.ALC.0000121651.75952.39 15100609

[8] Ford DE Kamerow DB Epidemiologic study of sleep disturbances and psychiatric disorders: an opportunity for prevention? Jama (1989) 262:1479–84. 10.1001/jama.262.11.1479 2769898

[9] Ohayon MM Prevalence of DSM-IV diagnostic criteria of insomnia: distinguishing insomnia related to mental disorders from sleep disorders. J Psychiatr Res (1997) 31:333–46. 10.1016/S0022-3956(97)00002-2 9306291

[10] Dong H Yang M Liu L Zhang C Liu M Shen Y Comparison of demographic characteristics and psychiatric comorbidity among methamphetamine-, heroin-and methamphetamine-heroin co-dependent males in Hunan, China. BMC Psychiatry (2017) 17:183. 10.1186/s12888-017-1346-7 28499448 PMC5427618

[11] Liao Y Tang J Liu T Chen X Luo T Hao W Sleeping problems among Chinese heroin-dependent individuals. Am J Drug Alcohol Abuse (2011) 37:179–83. 10.3109/00952990.2010.535580 21438798

[12] Tang J Liao Y He H Deng Q Zhang G Qi C Sleeping problems in Chinese illicit drug dependent subjects. BMC Psychiatry (2015) 15:28. 10.1186/s12888-015-0409-x 25884573 PMC4337091

[13] Alimoradi Z Lin C-Y Broström A Bülow PH Bajalan Z Griffiths MD Internet addiction and sleep problems: a systematic review and meta-analysis. Sleep Med Rev (2019) 47:51–61. 10.1016/j.smrv.2019.06.004 31336284

[14] D’Souza L Negahban MB Instagram virtual network addiction and sleep quality among students pursuing a speech and hearing course. Interdiscip J Virtual Learn Med Sci (2019) 10:1–10. 10.5812/IJVLMS.89059

[15] Schierenbeck T Riemann D Berger M Hornyak M Effect of illicit recreational drugs upon sleep: cocaine, ecstasy and marijuana. Sleep Med Rev (2008) 12:381–9. 10.1016/j.smrv.2007.12.004 18313952

[16] Quera-Salva M Orluc A Goldenberg F Guilleminault C Insomnia and use of hypnotics: study of a French population. Sleep (1991) 14:386–91. 10.1093/sleep/14.5.386 1759090

[17] Arber S Bote M Meadows R Gender and socio-economic patterning of self-reported sleep problems in Britain. Soc Sci Med (2009) 68:281–9. 10.1016/j.socscimed.2008.10.016 19026480

[18] Liao Y Xie L Chen X Kelly BC Qi C Pan C Sleep quality in cigarette smokers and nonsmokers: findings from the general population in central China. BMC Public Health (2019) 19:808. 10.1186/s12889-019-6929-4 31234809 PMC6591832

[19] Romanelli F Smith KM Clinical effects and management of methamphetamine abuse. Pharmacother: J Hum Pharmacol Drug Ther (2006) 26:1148–56. 10.1592/phco.26.8.1148 16863490

[20] Howe RC Hegge FW Phillips JL Acute heroin abstinence in man: I. Changes Behav sleep. Drug Alcohol Depend (1980) 5:341–56. 10.1016/0376-8716(80)90160-X 7371499

[21] Young-McCaughan S Miaskowski C Definition of and mechanism for opioid-induced sedation. Pain Manage Nurs (2001) 2:84–97. 10.1053/jpmn.2001.25012 11710090

[22] Chen VC-H Ting H Wu M-H Lin T-Y Gossop M Sleep disturbance and its associations with severity of dependence, depression and quality of life among heroin-dependent patients: a cross-sectional descriptive study. Subst Abuse Treatment Prevention Policy (2017) 12:16. 10.1186/s13011-017-0101-x PMC5360025 28320448

[23] Tsai P-S Wang S-Y Wang M-Y Su C-T Yang T-T Huang C-J Psychometric evaluation of the Chinese version of the Pittsburgh Sleep Quality Index (CPSQI) in primary insomnia and control subjects. Qual Life Res (2005) 14:1943–52. 10.1007/s11136-005-4346-x 16155782

[24] Zhang B Wing Y-K Sex differences in insomnia: a meta-analysis. Sleep (2006) 29:85–93. 10.1093/sleep/29.1.85 16453985

[25] Steptoe A Peacey V Wardle J Sleep duration and health in young adults. Arch Internal Med (2006) 166:1689–92. 10.1001/archinte.166.16.1689 16983045

[26] Gangwisch JE Heymsfield SB Boden-Albala B Buijs RM Kreier F Pickering TG Short sleep duration as a risk factor for hypertension: analyses of the first National Health and Nutrition Examination Survey. hypertension (2006) 47:833–9. 10.1161/01.HYP.0000217362.34748.e0 16585410

[27] Miller MA Kandala N-B Kivimaki M Kumari M Brunner EJ Lowe GD Gender differences in the cross-sectional relationships between sleep duration and markers of inflammation: Whitehall II study. Sleep (2009) 32:857–64. 10.1093/sleep/32.7.857 PMC2706900 19639748

[28] Mayo LM Paul E DeArcangelis J Van Hedger K de Wit H Gender differences in the behavioral and subjective effects of methamphetamine in healthy humans. Psychopharmacology (2019) 236:2413–23. 10.1007/s00213-019-05276-2 PMC6695366 31165207

[29] He Y Zhai J Liu Y Association of methamphetamine use with depressive symptoms and gender differences in this association: a meta-analysis. J Subst Use (2020) 25:440–48. 10.1080/14659891.2020.1736659

[30] Eid RS Gobinath AR Galea LA Sex differences in depression: Insights from clinical and preclinical studies. Prog Neurobiol (2019) 176:86–102. 10.1016/j.pneurobio.2019.01.006 30721749

[31] Perez AY Kirkpatrick MG Gunderson EW Marrone G Silver R Foltin RW Residual effects of intranasal methamphetamine on sleep, mood, and performance. Drug Alcohol Depend (2008) 94:258–62. 10.1016/j.drugalcdep.2007.10.011 PMC2267907 18078723

[32] Lin S Chen C Ball D Liu H Loh E Gender-specific contribution of the GABA A subunit genes on 5q33 in methamphetamine use disorder. pharmacogenomics journal (2003) 3:349–55. 10.1038/sj.tpj.6500203 14569258

[33] Hellem TL Lundberg KJ Renshaw PF A review of treatment options for co-occurring methamphetamine use disorders and depression. J Addict Nurs (2015) 26:14. 10.1097/JAN.0000000000000058 25761159 PMC5510330

[34] Dluzen DE Liu B Gender differences in methamphetamine use and responses: a review. Gender Med (2008) 5:24–35. 10.1016/S1550-8579(08)80005-8 18420163

[35] Dolsen MR Harvey AG Life-time history of insomnia and hypersomnia symptoms as correlates of alcohol, cocaine and heroin use and relapse among adults seeking substance use treatment in the United States from 1991 to 1994. Addiction (2017) 112:1104–11. 10.1111/add.13772 PMC5407928 28127809

[36] Bootzin RR Stevens SJ Adolescents, substance abuse, and the treatment of insomnia and daytime sleepiness. Clin Psychol Rev (2005) 25:629–44. 10.1016/j.cpr.2005.04.007 15953666

[37] Deng Q Tang Q Schottenfeld RS Hao W Chawarski MC Drug use in rural China: a preliminary investigation in Hunan Province. Addiction (2012) 107:610–3. 10.1111/j.1360-0443.2011.03648.x PMC4596718 21906200

[38] Phillips BA Danner FJ Cigarette smoking and sleep disturbance. Arch Internal Med (1995) 155:734–7. 10.1001/archinte.155.7.734 7695462

[39] Kenney SR LaBrie JW Hummer JF Pham AT Global sleep quality as a moderator of alcohol consumption and consequences in college students. Addictive Behav (2012) 37:507–12. 10.1016/j.addbeh.2012.01.006 PMC4329778 22285119

[40] Tang S Tang W Meyers K Chan P Chen Z Tucker JD HIV epidemiology and responses among men who have sex with men and transgender individuals in China: a scoping review. BMC Infect Dis (2016) 16:588. 10.1186/s12879-016-1904-5 27765021 PMC5073436

[41] Zhao P Tang S Wang C Zhang Y Best J Tangthanasup TM Recreational drug use among Chinese MSM and transgender individuals: results from a national online cross-sectional study. PloS One (2017) 12:e0170024. 10.1371/journal.pone.0170024 28107391 PMC5249205

